# Feasibility Study on Cardiac Arrhythmia Ablation Using High-Energy Heavy Ion Beams

**DOI:** 10.1038/srep38895

**Published:** 2016-12-20

**Authors:** H. Immo Lehmann, Christian Graeff, Palma Simoniello, Anna Constantinescu, Mitsuru Takami, Patrick Lugenbiel, Daniel Richter, Anna Eichhorn, Matthias Prall, Robert Kaderka, Fine Fiedler, Stephan Helmbrecht, Claudia Fournier, Nadine Erbeldinger, Ann-Kathrin Rahm, Rasmus Rivinius, Dierk Thomas, Hugo A. Katus, Susan B. Johnson, Kay D. Parker, Jürgen Debus, Samuel J. Asirvatham, Christoph Bert, Marco Durante, Douglas L. Packer

**Affiliations:** 1 Mayo Clinic Translational Interventional Electrophysiology Laboratory, Mayo Clinic, Rochester, MN, USA; 2 Department of Biophysics GSI Helmholtzzentrum für Schwerionenforschung, Darmstadt, Germany; 3 Department of Cardiology, University of Heidelberg, Heidelberg, Germany; 4 Department of Radiation Oncology, Friedrich-Alexander University Erlangen-Nürnberg, Erlangen, Germany; 5 Helmholtz-Zentrum Dresden-Rossendorf, Institute of Radiation Physics, Dresden, Germany; 6 Heidelberg Ion-Beam Therapy Center (HIT), Heidelberg, Germany; 7 Trento Institute for Fundamentals Physics Applications (TIFPA-INFN), University of Trento, Trento, Italy

## Abstract

High-energy ion beams are successfully used in cancer therapy and precisely deliver high doses of ionizing radiation to small deep-seated target volumes. A similar noninvasive treatment modality for cardiac arrhythmias was tested here. This study used high-energy carbon ions for ablation of cardiac tissue in pigs. Doses of 25, 40, and 55 Gy were applied in forced-breath-hold to the atrioventricular junction, left atrial pulmonary vein junction, and freewall left ventricle of intact animals. Procedural success was tracked by (1.) in-beam positron-emission tomography (PET) imaging; (2.) intracardiac voltage mapping with visible lesion on ultrasound; (3.) lesion outcomes in pathohistolgy. High doses (40–55 Gy) caused slowing and interruption of cardiac impulse propagation. Target fibrosis was the main mediator of the ablation effect. In irradiated tissue, apoptosis was present after 3, but not 6 months. Our study shows feasibility to use high-energy ion beams for creation of cardiac lesions that chronically interrupt cardiac conduction.

Disturbances of the heart rhythm, cardiac arrhythmias, are a major source for morbidity and mortality worldwide. Arrhythmias occurring from the ventricles may lead to sudden cardiac death that is estimated to account for about 300,000 deaths in the United States per year^1^. Atrial fibrillation is an extremely common cardiac arrhythmia^2^. The prevalence of atrial fibrillation is on significant rise as populations are ageing, placing individuals at increased risk for stroke, comorbidities and mortality^3^.

Catheter ablation, which commonly uses radiofrequency energy or cryothermal technology, has also evolved into a powerful treatment option for atrial fibrillation^2^ and ventricular tachycardia^4^. Yet, success rates of catheter ablation in these diseases are still limited. This is because the source often cannot be eliminated applying these presently used energy sources to endo- and epicardial surfaces of the heart^5^. Furthermore, catheter ablation is linked to several complications, including interventional risks such as silent embolic events^6^,^7^, vessel or cardiac wall perforation^2^, and atrial-esophageal fistula formation^8^.

These pitfalls could be overcome by noninvasive use of charged particle beams. In fact, accelerated protons and heavier ions (generally carbon ions) deliver most of their energy in the distal region of their path in the tissues (Bragg peak)^9^. Beam penetration depth is controlled by the initial particle energy in the accelerator. Pencil-beam scanning by magnetic deflection can deliver the radiation dose conformal to arbitrarily shaped targets^10^. Favorable physical and biological characteristics of charged particles compared to X-rays justify their use in cancer therapy, where they are considered a cutting-edge technology^9^. Irradiation with carbon ions can be monitored by Positron (β^+)^ Emission Tomography (PET) as nuclear fragments of target and projectile yield β^+^-emitters with half-lives of up to about 20 min^11^.

We have recently provided initial data to ablate cardiac arrhythmias with ion beams in isolated Langendorff-perfused beating heart preparations^12^,^13^,^14^. Our working hypothesis is that high-energy charged particles can be used to create chronic lesions that locally interrupt cardiac conduction and therefore enable treatment of heart rhythm disorders completely noninvasively while sparing surrounding tissues.

In this first *in situ* heart feasibility study, we demonstrate ablation with chronic interruption of impulse propagation of several cardiac locations by using charged particle beams in a sham-controlled, large animal model without any required procedural access to the body.

## Results

### Study Design and Overview

Seventeen animals were randomized to irradiation of the atrioventricular junction (AVJ, n = 8), right superior pulmonary vein (RSPV) left atrial (LA) junction (n = 3), freewall left ventricle (LV, n = 3), and compared to sham-procedures (n = 3). Target contours for the respective ablation location were placed at the AVJ, LV freewall, and LA-RSPV junction (cf. methods). Baseline characteristics of the animals and relevant contouring and targeting parameters are depicted in Table 1. The mean baseline left ventricular ejection fraction was 73 ± 4%. The mean duration of follow-up was 20.3 weeks. All animals of all assigned dose and target groups stayed in sinus rhythm during irradiation.

### Atrioventricular Junction Ablation Group

#### Carbon Ion Beam Treatment Planning and In-Beam PET for Monitoring of Dose Deposition

**Figure 1:**
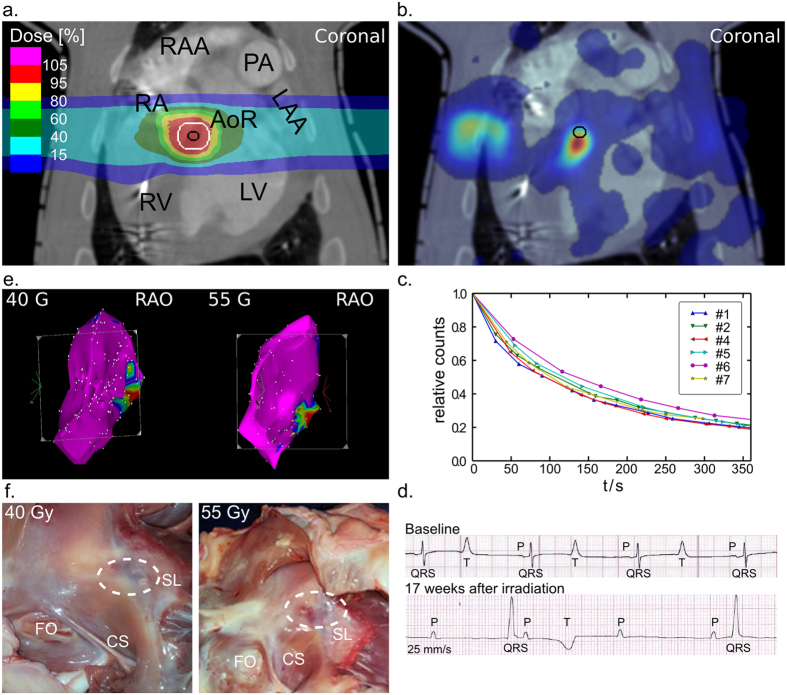
Application of Carbon Ions for Atrioventricular Junction Ablation. (**a**) Coronal view of a treatment plan for irradiation of the atrioventricular junction. Dose depicted as color-wash, the target contour is shown in black, the enlarged target contour in white. 100% corresponds to the prescribed dose of 55 Gy. (**b**) Image of positron emission tomography after 55 Gy of carbon, projected over the coronal plane of a contrast-enhanced CT scan; the target contour is shown in black. (**c**) Decay of the detected ß^+^-signal over the course of six minutes. (**d**) Surface ECG (25 mm/sec) before irradiation and 17 weeks after irradiation. (**e**) Right anterior oblique (RAO) projection of right atrial electroanatomical voltage maps obtained *via* an intracardiac catheter with point-by-point sampling after ablation. Voltage legend is shown next to the image; local voltage >1.0 mV depicted in magenta. Voltage <0.5 mV depicted in red. Other colors mark voltages in-between. (**f**) Right lateral views of lesion outcomes at the tricuspid annulus; dashed lines mark the respective lesion location. AoR = Aortic root; CS = Ostium of coronary sinus; FO = Fossa ovalis; LAA = Left atrial appendage; LV = Left ventricle; PA = Pulmonary artery; RA = Right atrium; RV = Right ventricle; SL = Septal leaflet of the tricuspid valve.

An exemplary planned 4D-dose deposition including beam rescanning (cf. methods) for ablation of the contoured AVJ lesion with 55 Gy is shown in Fig. 1a. To ensure delivery of >95% of the prescribed dose to the target, isotropic and anisotropic margins were added which also extended into the blood (target volumes see Table 1). Online PET-imaging during irradiation with 55 Gy showed strong β^+^-activity in the atrioventricular (AV) septum (Fig. 1b) and a lower signal along the beam’s entry channel. Deposition of 40 and 25 Gy resulted in a lower but evaluable PET response. Radioactive decay and biological washout led to a relatively fast β^+^-signal reduction over a time-course of six minutes (Fig. 1c).

#### Surface ECG and Decremental Pacing Outcomes after Carbon Ion Irradiation

An overview of the electrophysiological effects of irradiation is given in Fig. 1d,e. In two out of six animals, both irradiation with 55 and 40 Gy led to complete AV block with presence of a junctional escape rhythm (Fig. 1d), which developed up to seventeen weeks after irradiation (#1, 4; Table 2). One other animal of the 55 Gy dose group did not show significant change in AV conduction at termination of follow-up (#2; Table 2). In the animal that developed complete AV block following 40 Gy (#4), block was not persistent until the end of follow-up at six months. However, an increased Wenckebach-point in relation to the animal’s own baseline (#4, baseline: 260 ms *versus* post-irradiation: 570 ms, Table 2) was present. Over the course of six months of follow-up, no electrophysiological effect appeared in the two animals irradiated with 25 Gy (#7, 8; Table 2) and in sham-animals.

#### Electroanatomical Mapping and Correlation to Macroscopic Lesion Outcome after Irradiation

To describe extent of the lesion that led to AV block, electroanatomical voltage mapping was conducted after twenty-four weeks of follow-up. In the animal that developed AV block following 55 Gy, voltage mapping revealed a lesion area of 2.7 cm^2^ *versus* an area of 1.4 cm^2^ for the 40 Gy animal (Fig. 1e), concordant with macroscopically endocardial lesion dimensions in two planes (#1, 4; Table 2). Compared to sham-procedure animals, 55 and 40 Gy in these two animals reduced the mean bipolar voltage amplitude of all AVJ target location tag-points by 2.4 mV (#1; p < 0.0001, [n = 207]) and 2.0 mV (#4; p < 0.0001, [n = 246]), respectively. In the animal of the 55 Gy group without apparent effect, the lesion was misplaced into the posterior left ventricular outflow tract.

### Left Atrial Pulmonary Vein Junction Ablation Group

#### Treatment Planning and In-Beam PET for Monitoring of Dose Deposition

**Figure 2:**
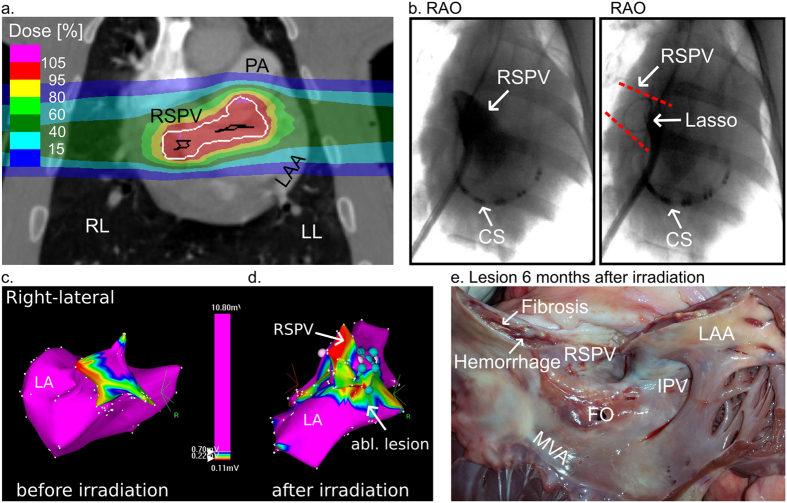
Impact of Carbon Ions on the Right Superior Pulmonary Vein Left Atrial Junction. (**a**) Coronal view of a treatment plan for irradiation of the right superior pulmonary vein (RSPV) left atrial (LA) junction. Details as described for Fig. 1a. (**b**) Right anterior oblique (RAO) view of RSPV venography and RAO of circumferential mapping catheter in the RSPV. Dashed red lines mark projection of RSPV. (**c**) Right-lateral projection of an endocardial electroanatomical voltage map from the left atrium (LA) and RSPV before irradiation. (**d**) Right-lateral projection of an electroanatomical voltage map of the LA and RSPV six months after irradiation with 40 Gy carbon ions. Voltage as in legend indicated; voltages >0.7 mV are depicted in magenta and voltage <0.2 mV in red. Other colors mark voltages in-between. (**e**) Macroscopic lesion outcome at the RSPV-LA junction. The RSPV is opened at its ostium at 12o’clock. Appreciate the macroscopically evident lesion with local hemorrhage and fibrosis. CS = multielectrode catheter in the coronary sinus. Circ. = the circumferential mapping catheter. Turquois dots = double potential. White dots = fragmented signals/area of slow conduction. FO = Transseptal puncture side in the fossa ovalis; LAA = left atrial appendage; MVA = Mitral valve annulus; RSPV = right superior pulmonary vein.

An exemplary coronal plane of a treatment plan for irradiation of the RSPV-LA junction is depicted in Fig. 2a. Despite the contoured lesion being located on the RSPV-LA junction, high-dose isodose lines extended into the RSPV muscular sleeve (Fig. 2a). During irradiation, PET showed only weak β^+^-activity (Fig. S1), likely due to the blood included in the target volume, that is quickly washed out.

#### Multielectrode Catheter Mapping after Carbon Ion Irradiation

Figure 2b displays a representative image of the circular multielectrode catheter positioned at the RSPV’s ostium. In one of the three studied animals (#12; Table 2), irradiation with 40 Gy led to almost complete disappearance of local antral atrial electrograms in the contoured target area.

#### Electroanatomical Mapping after Carbon Ion Irradiation

A voltage map of the LA and RSPV before irradiation is shown in Fig. 2c. In this animal (#12), voltage mapping after irradiation revealed a large change in local voltage tag points of the atrial myocardium close to the RSPV ostium (mean ∆: −1.7 mV; p < 0.0001, [n = 518]). A small posterior-inferior gap at the carina to the inferior PV was present (Fig. 2d). Extent of the LA ablation lesion over the roof and anterior mitral isthmus led to change in the myocardial activation sequence with late activation of the LA in this animal. In the other two animals, there was a statistically significant decrease in voltage at the RSPV-LA junction compared to baseline present (#13, 14; Table 2), however, this was notably smaller.

#### Macroscopic Lesion Outcome at Termination of Follow-up

The macroscopic lesion outcome at the RSPV-LA junction six months after irradiation is shown in Fig. 2e (#12). The main portion of the ablation lesion was located in the intended area. Nevertheless, the ablation lesion extended into myocardium of the PV muscle sleeve, left atrial roof, and LAA, reflecting areas included into the relatively large beam deposition margin (white contour Fig. 2a), added to the target contour (cf. methods). In the two other cases (#13, 14; Table 2), there was no macroscopically visible lesion at the RSPV-LA junction present.

### Outcomes in Ventricular Tissue–Freewall Left Ventricle Group

#### Carbon Ion Beam Treatment Planning and In-Beam PET for Monitoring of Dose Deposition

**Figure 3:**
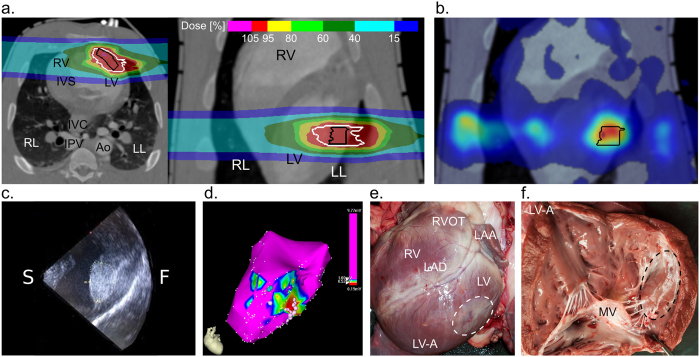
Outcomes after Left Ventricular (LV) Freewall Irradiation. (**a**) Transverse and coronal plane of a treatment plan with two lateral carbon beams (40 Gy). Details as described for Fig. 1a. (**b**) Anteroposterior view of by-product β^+^-activity during irradiation *via* PET, superimposed on the coronal plane of a contrast-enhanced CT scan. Black contour = target contour, β^+^-activity shown as color-wash. (**c**) Intracardiac ultrasound image of the LV freewall after irradiation, hyperechoic lesion area marked by dotted lines. (**d**) Left anterior oblique view of an endocardial voltage map of the LV 6 months after irradiation. As indicated in the legend, local voltage >1.0 mV is depicted in magenta. Voltage <0.5 mV in red. Other colors mark voltages in-between. White dots = fragmented potentials. (**e**) Macroscopic LV epicardial lesion outcome six months after irradiation with the dashed line marking the contoured target zone. (**f**) Endocardial lesion outcome six months after irradiation. Ao = Descending aorta; F = Freewall; LAA = Left atrial appendage; LAD = Left anterior descending coronary artery; LIPV = Left inferior pulmonary vein; LVOT = Left ventricular outflow tract behind the mitral valve leaflet; LL = Left lung; IVC = Inferior vena cava; LV = Left ventricle; LV-A = Left ventricular apex; RIPV = Right inferior pulmonary vein; RL = Right lung; RV = Right ventricle; S = Septal site.

Target contours were located at the LV freewall; a treatment plan, depicting transverse and coronal planes, with two lateral beam entry fields is shown in Fig. 3a. Table 1 shows the mean target volume including margins to compensate for contractile cardiac motion. Irradiation of the LV freewall induced strong β^+^-activities captured in PET scanning in all animals (Fig. 3b) with pronounced washout due to myocardial perfusion.

#### Electroanatomical Mapping and Intracardiac Echocardiography for *in-vivo* Lesion Characterization

All three irradiated animals showed some beam effects in the LV target location. Table 2 (#15–17) displays outcomes in LV tissue after thirteen and twenty-five weeks of follow-up. In two cases, examined after thirteen weeks (#15, 16), intracardiac echocardiography displayed a circumscribed area of hyperechoic myocardium at the LV freewall (Fig. 3c). Here, endocardial mapping revealed no statistically significant decrease in mean LV target voltage compared to baseline (#15, 16; Table 2). Epicardial mapping, however, showed clustering of fragmented potentials within the target location in one of these animals (#15; Table 2). In the animal from this group followed until twenty-five weeks after irradiation (#17), lesion appearance on intracardiac echocardiography was similar compared to thirteen weeks and endocardial mapping revealed decreased voltage within the target location (Fig. 3d) compared to baseline (mean baseline: 4.0 ± 2.3 *versus* post-irradiation: 0.6 ± 0.3; p < 0.0001, [n = 391]).

#### Macroscopic Lesion Outcome

Macroscopic epi- and endocardial lesion outcome is depicted in Fig. 3e,f. There was notable mid-myocardial as well as epicardial scar.

#### Lesion Histology (H&E, Mallory Trichrome) and Markers of Apoptosis Lesion Morphology

**Figure 4:**
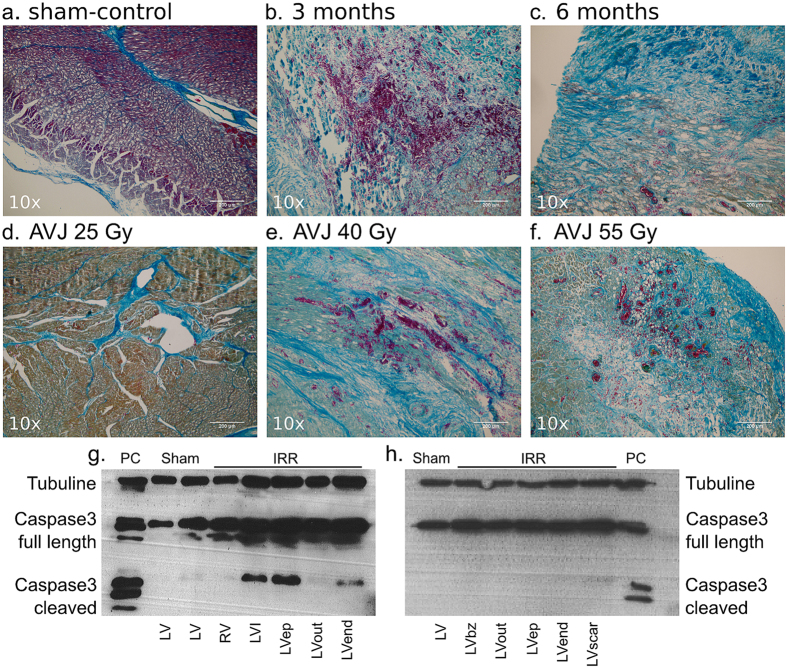
Mallory Trichrome Staining of Ablation Lesions, and Apoptosis Outcomes. (**a**) Sham-control (**b**) Target tissue three month after 40 Gy carbon ion irradiation with marked hemorrhage, inflammation, and early stage fibrosis, (**c**) Target tissue six months after carbon ion irradiation, showing later stage fibrosis. (**d–f**) Comparison of myocardial lesion outcomes for 25, 40, and 55 Gy of carbon ions six months after irradiation for the atrioventricular junction ablation group. (**g**) Western blot for cleaved caspase-3 a marker for apoptosis; signals for cleaved caspase-3 were positive in myocardium three months after irradiation, whereas no signals were observed six months after irradiation (**h**). Bz = borderzone; ep = Epicardium; IRR = Irradiated tissue; LV = Left ventricle; PC: positive control HaCaT (Lysats of HaCaT cells five days after irradiation with 10 Gy of X-ray), I = infield; Out = Outfield; RV = Right ventricle.

Compared to sham-animals, strong target fibrosis, cardiomyocyte disarray, and hemorrhage (Fig. 4b) was present in all animals with present blockage and macroscopically induced lesions (Table 2). Three months after irradiation, ablation lesions were marked by a higher degree of hemorrhage, inflammation, and early fibrosis than lesions six months after irradiation (Fig. 4b,c).

#### Dose Dependency

A comparison for the three different doses applied to the AVJ, namely 25, 40, and 55 Gy, is shown in Fig. 4d–f. Twenty-five Gy lead to minor fibrotic changes but, nevertheless, clearly led to manifold changes in the target tissue (cardiomyocyte disarray, apoptosis) while both 40 and 55 Gy in successful cases led to a strong fibrotic response as described above.

#### Tissue Apoptosis

Cleaved caspase-3 was only present in irradiated tissue after three months of follow-up, whereas lesions were negative for cleaved caspase-3 after six months of follow-up (Fig. 4g,h).

### Irradiation Toxicity during Follow-up

No damage was observed in the tissues of the esophagus, trachea, or phrenic nerves. Coronary arteries did not show a reaction during six months of follow-up. There was no statistical significant change in left ventricular ejection fraction between irradiated animals of all groups and sham-animals (mean ∆irradiation group: 2.4 ± 8.3 *versus* mean ∆sham-group: 3.3 ± 6.6; p = 0.81) or between sham-animals and LV freewall irradiation group present (Table 1). There was also no skin reaction in the beam’s entry channel noted in any animal.

## Discussion

This feasibility study indicates that: (i.) Heavy ion beams delivered by the raster scanning technique can be successfully used for ablation of myocardium with chronic interruption of impulse propagation. (ii.) In-beam PET is an accurate means for online verification of ion beam range and dose-deposition of these small, moving, and highly perfused cardiac targets. (iii.) Target doses of 40–55 Gy induce complete blockage of impulse propagation; such blockage is to be expected between thirteen to seventeen weeks after irradiation. (iv.) Ultrasound imaging can be used to depict created ablation lesions. (v.) Target fibrosis is the main mediator of interruption of cardiac impulse propagation, while multiple non-destructive structural and subcellular changes present at lower doses also affect arrhythmogenesis^15^,^16^.

Despite application of charged particles in non-cancer diseases being completely novel^17^, carbon ion beams to date have been applied as cutting-edge technology for treatment of malignant tumors in more than 15,000 patients worldwide^18^,^19^. The physical properties of charged particle beams–inverted dose-profile with major energy release at a specific tissue depth at the end of the particle range (Bragg peak)–enable irradiation of deep seated target volumes with significant sparing of surrounding structures when compared to state-of-the-art X-ray treatments^20^. In case of catheter-free arrhythmia ablation, this translates into decrease in dose-exposure of myocardium as well as mediastinal structures^21^. In addition to irradiation of very large myocardial volumes, studies using radiation therapy with photon beams for catheter-free isolation of the RSPV have created accidental heart block and fistulas of mediastinal organs in the porcine model^22^.

In this study, it took between thirteen to seventeen weeks for the electrophysiological endpoint to occur. There was no difference in that time-course between animals irradiated with 40 Gy and animals irradiated with 55 Gy; the time-course to occurrence of AV conduction block was longer than previously described using X-ray beams^23^,^24^. However, these data derive from a non-arrhythmic animal model and are based on conduction block only. Antiarrhythmic effects of ion beams in cardiac tissue may be multifactorial and therefore the time-course of true antiarrhythmic effects remains unclear. When using charged particles, the irradiated tissue volume is notably smaller and the conformity to the target volume is better than with photons, providing biophysical rationale for different cellular responses. Although our group has shown in isolated beating hearts that an acute electrophysiological effect may be seen with even higher dosing^12^, the present study does not show acute beam effects on cardiac conduction, but does show chronic effects within the applied dose range. Outcomes in this study were not concordant within each dose and target group. The reason for the relatively low success rate of 33% in AV and RSPV-LA junction ablation is likely to be multifactorial; three animals in the AVJ ablation group died of device-related infection and thus effects in these animals could not be evaluated in a similar fashion as in animals from the other groups. In one animal the lesion was misplaced by 5 mm in beam direction. This could have, among other factors, have been caused by a change in cardiac volume status, leading to a miscalculated position of the Bragg Peak. Range uncertainty especially for moving targets is a major issue in particle therapy. State-of-the-art imaging and matching capabilities such as cone beam CT are becoming clinically available for heavy ion therapy and would have facilitated positioning and matching of the treatment planning CT^25^, making placement of intracardiac markers unnecessary and increasing procedural efficacy. Technical capabilities were also restricted to a four degrees-of-freedom treatment table, instead of six degrees-of-freedom-robotic positioning table that is available at ion beam centers using the latest technology. Lastly, motion compensation for scanned particles is challenging^26^ and even though considerable care was taken to suppress the effects of cardiac contraction on the dose distribution (cf. methods), we cannot fully exclude residual dosing errors.

In-beam PET-imaging successfully verified ion beam range and irradiation of the respective target location. However, in this applied setup no subsequent corrections to the beam range or position were possible. In a single fraction treatment, PET imaging right after application of a relatively smaller amount of dose could be a solution to make early adjustments to ion beam position and/or ion range possible^27^. Strong washout in these highly perfused target volumes was observed, underlining favorable properties of online in-beam PET for this application. However, combination of PET with CT or MRI as it is done offline, immediately following the treatment of different tumors^28^ provides real-time high-resolution anatomy, but at the expense of increased washout and loss of decays with a too short half-life.

Apoptotic markers were found positive after three months, but negative after six months. Activation of the apoptotic cascade, particularly of endothelial cells, is a known response to ionizing radiation in tumorous tissue^29^. In the heart, ionizing radiation lesions have also been found to start with endothelial vascular damage, ultimately leading to myocardial fibrosis^30^. To our knowledge, there is no previous data on the role and the time-course of activation of the apoptotic cascade as response to ionizing radiation of the *in situ* heart. However, the decisive role of apoptosis in acute myocardial infarction^31^ and subsequent scar remodeling is quite well established^32^. Dose volume relationship for cardiac targets and dependence on ablated anatomical location remains unclear. Observed differences between endo- and epicardial fibrosis could reflect biological underpinnings such as different vascular density, but could also purely be related to physical targeting issues.

Arrhythmia ablation without physically accessing the body has been tried with other less focused energy sources^23^,^33^ and has tremendous clinical implications. It would eliminate the side effects and the risks of introducing and ablating with catheters in the central circulation, including the formation of blood clots, embolization, and perforation. Ablation for ventricular arrhythmias often fails because of limited accessibility of the arrhythmia substrate in the ventricles using standard techniques^34^,^35^. Ionizing irradiation does have long-term side effects that need to be carefully evaluated in further studies with longer follow-up and risk modeling. Toxicity data for high-dose irradiation of small cardiac volumes is scarce. This study shows acute safety when careful treatment planning is conducted, nevertheless, evaluation of long-term safety requires careful future studies. Theoretically, the physical properties of ion beams will allow localized, volume-conformal irradiation of arrhythmogenic sources at any given myocardial depth. This is not limited to carbon ions, but should also be valid for lighter ions with similar focusing capabilities that can even be accelerated using cyclotrons. Integrating particle beam technologies with cardiac MRI, CT, and body surface arrhythmia mapping^36^ would equip us with the necessary technology to achieve focused, completely noninvasive arrhythmia ablation. High-energy particle beams have potential as a new and precise means for external arrhythmia elimination without any required procedural access to the body and without intrinsic restriction of tissue penetration for this energy-source.

## Methods

All animal procedures and irradiation were approved by the regional board of the state of Baden-Württemberg, Karlsruhe, Germany (approval number G-7/14) as well as the committees of the Helmholtz Centre for Heavy Ion Research (GSI), Darmstadt, Germany. All animal procedures were carried out in accordance with the ‘German Law for Animal Research’ (Tierschutzgesetz) and with the NIH Guide for the Care and Use of Laboratory Animals as well as the guidelines established by the Mayo Foundation animal care and use committee.

### Study Design and Randomization to Target and Dose Groups

Seventeen pigs (*sus scrofa domestica*) of either sex (weight of 30–35 kg, age ~10 weeks) were randomized to irradiation of one of three targets or to a sham-irradiated control group (n = 3). Eight animals were assigned to irradiation of the AVJ. Here, in addition, randomization to dose (25 [n = 2], 40 [n = 3], and 55 Gy [n = 3]) was performed. Three pigs were randomized to irradiation of the RSPV-LA junction (40 Gy) and three animals to LV freewall irradiation (40 Gy). All animals received a baseline study, assessing pertinent cardiac parameters for later detailed evaluation of beam effects on myocardium. None of the information from this study was integrated and used for the actual guidance of beam delivery, which only relied on the CT image and the here extracted cardiac motion data. Three to six months after single fraction irradiation, animals underwent follow-up evaluation and were euthanized, tissues were harvested, and additional experiments were conducted as described below. All endpoints were prospectively selected.

In ion beam therapy for cancer, models predict the relative biological effectiveness (RBE) with the goal of delivering a biologically effective dose of carbon ions in Gy (RBE) with the same effect as a physical dose of photons in Gy. Due to the highly localized energy deposition of carbon ions leading to complex DNA damage, RBE values for carbon for typical dose ranges of 2–4 Gy/fraction are in the order of 3^37^. The RBE is dose-dependent; for very high single fraction doses used here, the RBE is likely considerably closer to 1^38^. RBE-models were established for cancerous cell killing, while the endpoint of influencing cardiac electrophysiology is completely unstudied. Therefore, we decided to use physical doses for the carbon beam treatment planning.

### Anesthesia and Surgical Care for Invasive Baseline and Follow-up Studies

Anesthesia was induced using an intramuscular injection of ketamine (100 mg/kg, Roche, Grenzach-Wyhlen, Germany), azaperone (2 mg/kg, Janssen Pharmaceutica, Beerse, Belgium), and midazolam (0.5 mg/kg, Roche, Grenzach-Wyhlen, Germany). In case of electrophysiological studies, after intubation, animals were maintained on 1–3% inhaled isoflurane (Baxter, Unterschleißheim, Germany) for anesthesia. ECG was monitored using four surface electrodes. Invasive arterial blood pressure, digital pulse oximetry, and end-tidal CO_2_ were constantly monitored. Analgesia after the procedure was achieved using morphine s.c. (1 mg/kg, Pfizer, NY, USA).

### Baseline Electrophysiological Study

Twelve-lead ECGs were obtained. Transthoracic echocardiography (Philips Healthcare Sonos 5500, Hamburg, Germany) was performed in a supine position. Skin was prepped using Braunol^®^ (B. Braun, Melsungen, Germany). For vascular access, cut-downs with vessel preparation for placement of introducer sheaths in the left/right external jugular vein and the right/left femoral arteries and veins were performed. A 7Fr decapolar catheter was advanced into the coronary sinus (CS) from the external jugular vein for electrogram recordings. All catheterization was performed under fluoroscopic guidance (Siremobil Combact L, Siemens Healthcare, Forchheim, Germany). For ultrasound imaging during the procedure, an intracardiac echocardiography (ICE [Acuson, Cypress, Mountain View, CA, USA]) with a 10Fr 5.5–10 MHz probe was used. For LA and LV access, a transseptal puncture was performed under ICE guidance using a BRK^TM^ needle (St. Jude Medical, St. Paul, MN, USA) through a SL1 sheath. Electroanatomical mapping was performed using a mapping system (Carto XP, Biosense Webster, Inc., Diamond Bar, CA, USA). Angiograms of right and left coronary arteries were performed using an AL-1 catheter (Cordis, Johnson and Johnson^®^, USA). One intracardiac marker was implanted (right atrial appendage) with an endovascular technique, serving as fiducial during biplane X-ray positioning for irradiation (Quick Clip 2, 8 mm × 2 mm, Olympus, Shinjuku, Japan).

#### Atrioventricular Junction Ablation and Sham-Group

Electroanatomical mapping with creation of three-dimensional, voltage, and activation maps was performed. A Navistar mapping catheter with a 3.5 mm distal tip electrode, 2.0 mm ring electrode, and 1 mm interelectrode distance was used (Biosense Webster, Inc., Diamond Bar, CA, USA). Around 200 points were sampled (fill-threshold <15 mm; high-density map) per chamber. Intracardiac signals were sampled at 1 kHz and filtered at 30–250 Hz (Bard medical, Covington, GA, USA). Bipolar voltage of endocardial target tag-points before and after irradiation was compared. Atrial signals of coronary sinus (CS) 1–2 were used as reference electrogram. Function of the AVJ was assessed using the work-flow described by Bunch *et al*.^39^. Antegrade decremental pacing was performed from the high right atrium, beginning at a cycle length 20 ms shorter than the sinus rate, down to 200 ms. The cycle length at which Wenckebach block occurred was established. AVJ ablation and sham-animals underwent dual chamber pacemaker implantation at the end of the baseline study. Preparation of the external jugular vein was performed. Two 7Fr active fixation pacing leads were introduced through two small incisions in the vessel wall. Atrial leads were placed in the right atrial appendage and right ventricular leads were placed in the right ventricular apex. Leads were connected to a pacemaker unit (Medtronic Inc., Minneapolis, MN, USA) placed in a subcutaneous pocket in the neck. Pacemakers were programmed in the DDI mode with a lower pacing rate of 60 bpm.

#### Pulmonary Vein Isolation Group

A steerable 8Fr sheath was used for LA mapping (Agilis, St. Jude Medical, St. Paul, MN, USA). A 7Fr decapolar circular mapping catheter (Biosense Webster, Palo Alto, CA, USA) was advanced into the RSPV-LA junction for recording of PV potentials. Recordings were obtained in sinus rhythm and during pacing. Electroanatomical mapping of the LA, RSPV-LA junction, CS, and right atrium adjacent to the RSPV was conducted. The atrial electrogram from CS 1–2 was used as reference. Bipolar voltage of endocardial target tag-points before and after irradiation was compared.

#### Left Ventricular Ablation Group

LV electroanatomical mapping was performed using a retrograde transaortic and/or transseptal approach. The surface QRS complex was used as mapping reference. Percutaneous epicardial access was obtained using a 6 inch, 17 gauge Tuohy needle inserted through a small sub-xiphoidal incision under fluoroscopic guidance^40^. After guidewire access to the epicardial space was confirmed, an 8Fr introducer sheath was inserted over the wire and positioned in the pericardial sac. Electroanatomical mapping of the epicardial surface of the LV was performed. Bipolar voltage of endocardial target tag-points before and after irradiation was compared.

### Sedation and Ventilation during Computed Tomography and Carbon Ion Irradiation

Sedation was induced as described above. Sedation during CT scanning and irradiation was maintained through intravenous infusion of propofol (10 mg/cc; 0.25–0.30 mg/kg/min, Diprivan, Fresenius, Bad Hoburg, Germany) without need for additional paralytic use for ventilator and breath-hold compliance. Oxygenation was maintained through intermittent-positive pressure ventilation (IPPV, Evita XL, Draeger, Lübeck, Germany).

### Four-Dimensional Computed Tomography Acquisition

Sedated animals were immobilized using a vacuum mattress and thermoplastic mask for reproducible positioning (Fig. S3). The CT reference point (calibrated room laser) was marked on the mask with radio-opaque markers and tattooed on the skin. Cardiac-gated (4-dimensional [D]), native and contrast-enhanced CT scans (10 phases) were acquired using a 64 row Siemens Somatom Definition Flash scanner (Siemens Healthcare, Forchheim, Germany). Scans were acquired at end-expiration using a 25 sec. respirator breath-hold remotely controlled through a Labview software interface (National Instruments, Austin, TX, USA). For enhanced scans, 50cc contrast medium were injected (4cc/sec, 8–10 sec. delay, Omnipaque 350 mg I/cc, GE Healthcare, USA). A field of view of 400 mm and 1 mm slice thickness was reconstructed. The native 4D-CT protocol was validated against the standard planning CT protocol of the Heidelberg Ion Beam Therapy Center (HIT) for correct HU and resulting ion beam ranges.

### Target Definition and Contouring in Computed Tomography Images

CT contouring to define specific structures was performed using Syngo^®^ PRT Planning (VC11B, Siemens AG, Erlangen, Germany) and Eclipse^TM^ (Varian medical, Palo Alto, CA, USA) treatment planning software as described^41^. Briefly, a 5 mm sphere was contoured for AVJ ablation. RSPV-LA junction and LV freewall ablation lesions were defined by polygons in the transverse CT slices. In addition, organs and structures at risk (OAR) such as the esophagus, trachea, aorta, and coronary arteries, were demarked on the 0% R-R phase of the 4D-CT.

### Four-Dimensional Carbon Ion Beam Treatment Planning

Treatment planning was conducted using GSI’s treatment planning software TRiP4D^42^. A treatment plan for scanned charged particles consists of sets of pencil beams of the same energy or range in water (iso-energy slices, IES). Pencil beams were arranged on a regular grid with 2 mm lateral distance. Along the beam axis, IES were spaced 3 mm apart in water. A so-called ripple filter broadened the Bragg peak to approximately 3 mm, so that homogeneous doses could be applied^43^. Through optimization, particle numbers were assigned to each pencil beam, so that the whole plan conforms with dose prescription to target and OARs.

To extend contours and doses across the 4D-CT phases, deformable image registration (DIR) was performed and yielded a voxel-to-voxel map between reference (0% R-R) and remaining motion phases (Plastimatch version 1.15.17)^44^. Quality assurance of registration was conducted by visualization of the vector field, false color, checkerboard images, propagation of contours, and the Jacobian matrix^45^. DIR was computed on the contrast-enhanced 4D-CTs but applied to native images, as contrast agent falsifies ion range information^46^. Target volumes of AVJ and PVI were isotropically expanded by 5 mm to account for setup errors. For LV freewall irradiation, 2 mm + 2% range margin (target depth uncertainty) was added. Using DIR, volumes were propagated to each phase and field-specific target volumes, including target motion and motion-induced range changes were obtained for all 4DCT phases (planning target volume = PTV)^47^. In scanned particle therapy, interference between the scanning motion of the beam and target motion leads to major dose errors (interplay)^48^. Delivery margins do not help against interplay, but repeated irradiation of each IES (rescanning) leads to averaging and a homogeneous target dose^49^. Four-dimensional treatment planning studies were conducted for all cases to ensure safe and appropriate delivery parameters despite contractile motion. Interplay was simulated using a detailed description of irradiation timing and several ECG tracings with variable heart rates^42^.

Using the ECG, the appropriate 4D-CT phase was determined for each pencil beam, allowing 4D-dose calculation. Total dose was accumulated in a reference phase using DIR. In these simulations, 15 slice-by-slice rescans were sufficient for motion compensation. Acceptable dose to the target was assumed if 95% of the contoured target received >95% of the prescribed dose (>90% including safety margins) in 4D simulations. For faster irradiation, full rescan cycles were only applied to the dominant, high energy IES and were reduced for lower energies.

Doses to OAR were studied. Constraints for trachea, aorta, and esophagus derived from single fraction lung cancer radiotherapy^50^. For AVJ and LV irradiation, two fields were individually optimized to a homogeneous dose, leading to robust irradiation without gradients. For RSPV-LA junction, irradiation fields were optimized simultaneously permitting gradients between fields in the target to better spare the esophagus (intensity modulated particle therapy IMPT). For one animal the RSPV-LA junction dose had to be reduced to 30 Gy for sufficient sparing of the esophagus. In simulations, a margin size of 5 mm for AVJ and RSPV-LA junction was determined as the minimally acceptable margin, ensuring target coverage after repositioning, without sacrificing dose constraints to OARs.

### Quality Assurance for Charged Particle Irradiation

For quality assurance (QA), all treatment plans were irradiated into a water phantom prior to actual delivery. Dose was measured by pinpoint ionization chambers attached to an industrial robotic arm^51^. Static and dynamic QA was performed, with the arm being stationary or moving in the motion trajectory extracted from the cardiac 4DCT. Measured dose was compared against corresponding (4D) calculations.

### Charged Particle Irradiation

Irradiation was carried out at the GSI Helmholtz Centre for Heavy Ion Research, Darmstadt, Germany where a single horizontal beam line is used. Pigs were immobilized in the same device used for treatment planning imaging (Fig. S2). Masks and skin tattoos were aligned to the room-laser. Positional concordance was ensured using repeated matching of two orthogonal X-ray images to CT-derived digitally reconstructed radiographs followed by repositioning using the treatment table (four degrees of freedom). Pencil beam scanning followed the raster-scanning technique with rescanning. Beams were irradiated using breath-holds at expiration. The average pencil beam focus size in the isocenter was 6.5 mm (full width at half maximum).

### In-Beam Positron Emission Tomography (PET) Imaging

For monitoring of dose deposition, detection of β^+^-activity resulting from fragmentation of target and projectile nuclei was performed. Scans were obtained during and immediately after irradiation to study washout phenomena. GSI has a dedicated two-head, in-beam PET camera integrated into the treatment side^11^,^52^. For imaging, detector heads (CTI PET Systems Inc., Knoxville, TN, USA) were positioned above and below the treatment couch. PET was conducted in expiration for the first delivered field to avoid superimposed activity from subsequent irradiation^52^. Presented PET images display activity produced through half of the total delivered dose. PET activity was superimposed on the reconstructed treatment planning contrast-enhanced CT for better visualization of cardiac anatomy.

### Follow-up after Irradiation

Animals were followed for up to six months after irradiation. Twelve-lead ECGs and device interrogations were performed after 4, 8, 13, and 24 weeks. At follow-up termination, animals underwent repeat electrophysiological study as described above. Animals were euthanized at the end of the last invasive follow-up procedure through intracardiac potassium injection.

### Pathological Examination

Heart, lungs, trachea, phrenic nerves, and esophagus were removed *en* block with the pericardium intact. Pericardial fluid was drained and collected. Gross pathological findings were assessed and documented. Triphenyltetrazolium chloride was used to delineate the ablation lesions. Skin biopsies of the beam entry channel (*in-field*) and control samples (*out-field*) were obtained at baseline and during the follow-up procedures and processed for evaluation.

### Histological Examination

Histological analysis was conducted on all animals. Samples were fixed in 4% formaldehyde and processed as described^53^. Subsequently, samples were wax embedded and cut with a microtome. Cut sections (5 μm) were stained with Hematoxylin & Eosin and Mallory Trichrome for evaluation using light microscopy.

### Protein Extraction and Western Blotting

Protein lysation and Western blotting were conducted according to our protocols^53^,^54^, on all specimens. Used antibodies were anti-caspase-3, anti-tubuline (Sigma Aldrich), and horseradish peroxidase-conjugated secondary antibodies (GE Healthcare Life Sciences). Protein expression was visualized using enhanced chemiluminescence (ECL, Amersham Biosciences) and detected on films (ECL-Hyperfilm, Amersham Biosciences).

### Statistical Analysis

Statistical analyses were performed using IBM’s SPSS software (version 23.0). Three animals deceased from device-related infection; follow-up data of these animals was included until the available date of death and all other data was included. Continuous variables are presented as mean ± standard deviation or in case of skewed distribution as median and range. Comparison of voltage mapping data for each animal before and after irradiation was performed using a two-sided t-test (Table 2) or Mann-Whitney’s U test depending upon distribution of variables. The mean target tag-point voltage at baseline was compared to mean target tag-point voltage after irradiation. A p-value < 0.05 was considered to indicate statistical significance.

## Additional Information

**How to cite this article**: Lehmann, H. I. *et al*. Feasibility Study on Cardiac Arrhythmia Ablation Using High-Energy Heavy Ion Beams. *Sci. Rep.* **6**, 38895; doi: 10.1038/srep38895 (2016).

**Publisher's note:** Springer Nature remains neutral with regard to jurisdictional claims in published maps and institutional affiliations.

## Supplementary Material

Supplementary Dataset 1

## Figures and Tables

**Table 1:** Study population general metrics, results for global cardiac function and treatment planning parameters for all dose groups and targets.

Study Group								
		All Pigs (n = 17)	Sham-control (n = 3)	AVJ 25 (n = 2)	AVJ 40 (n = 3)	AVJ 55 (n = 3)	RSPV-LA junction (n = 3)	Freewall LV (n = 3)
General Metrics	Male sex, n (%)	13 (76)	3 (100)	2 (100)	1 (33)	3 (100)	2 (66)	2 (33)
	Mean weight at imaging, kg	33.8 ± 3.4	32.5 ± 4.6	35.1 ± 3.1	35.5 ± 1.2	31.6 ± 1.7	36.5 ± 2.6	33.6 ± 2.9
	Mean weight at irradiation, kg	34.2 ± 3.2	n.a.	30.2	36.4 ± 1.7	32.0 ± 1.7	35.0 ± 3.0	34.1 ± 3.9
	Mean weight gain at 6 months, kg	39.8 ± 14.0	36¥	54.3 ± 8	32.8 ¥	50.3 ± 1.0	35.0 ± 2.1	47¥
	Mean duration of follow-up, weeks	20.3 ± 5.7	18.7 ± 5.6	24.4 ± 0.1	17.3 ± 5.3	20.3 ± 5.6	24.5 ± 0.1	16.6 ± 5.6
	Mean time from CT to Irradiation, days	10.9 ± 3.4	n.a.	13.5 ± 1.5	16.0 ± 1.4	14.0 ± 2.5	8 ± 0	9.3 ± 0.5
Cardiac Function	Baseline LVEF (%)	73 ± 4	70 ± 2	77 ± 6	72 ± 4	78 ± 1	74 ± 1	73 ± 5
	LVEF (%) at 6 months	76 ± 6	73 ± 8	75 ± 4	79¥	79 ± 1	72 ± 2	78 ± 7
	Baseline E/A wave ratio	0.91 ± 0.5	0.66 ± 0.02	0.54 ± 0.01	1.0 ± 0.5	0.7 ± 0.2	1.1 ± 0.2	1.3 ± 0.8
	E/A wave ratio at 6 months	0.8 ± 0.2	0.74 ± 0.1	1.2 ± 0.1	0.6¥	0.6 ± 0.01	0.6 ± 0.04	1.0 ± 0.3
Irradiation Parameters	Mean delineated target volume (cc)	n.a.	n.a.	0.5	0.5	0.5	0.9 (0.8–1.1)¶	2.3 ± 0.3
	Mean target volume + margins (cc)	n.a.	n.a.	1.8 ± 0.1	1.8 ± 0.1	1.8 ± 0.1	14.9 ± 1.8	n.a.
	Mean planning target volume (PTV, cc)	n.a.	n.a.	3.7 ± 0.3	3.4 ± 0.5	3.9 ± 1.0	23.2 ± 1.7	8.9 ± 1.8
	Iso-energy slices, n (IES)	n.a.	n.a.	11.5 ± 1.5	9 ± 1.4	8.3 ± 0.5	20 ± 1	14
	Beam energies, MeV	n.a.	n.a.	172–211	175–208	183–212	129–233	112–186
	Mean treatment time (min)	n.a.	n.a.	15.7	7.3	10.8	23.4 ± 8.1	23.9 ± 8.6

The mean planning target volume corresponds to the irradiated volume. AVJ = atrioventricular junction ablation; RSPV-LA junction = right superior pulmonary vein left atrial junction; LVEF = Left Ventricular Ejection Fraction; MeV = Megaelectronvolt; min = minutes; n.a. = not applicable; ¥ = Data only available for one animal. Data are depicted as mean ± standard deviation. ^¶^Median (range).

**Table 2:** Overview of the outcomes for all irradiated animals and the sham group.

Animal #	AV junction ablation Dose (Gy)	Mapping Outcome (cm^2^)–tag points with Voltage ≤ 0.3 mV	Macroscopic Lesion Surface (cm^2^)	Macroscopic Lesion	Lesion in Histology	∆Wenckebach end Follow-up (ms)	Follow-up (weeks)
1	55¥	2.9	2.5	Yes	Yes	n.a. 3° AVB	24.3
2	55	0	1.7¶	Yes¶	¶	10	24.2
3	55	†	0	†	†	†	12.4
4	40¥	1.3	1.0	Yes	Yes	310	24.3
5	40	†	0	†	†	†	11.6
6	40	†	0	†	†	†	16.0
7	25	0	0	No	Yes	10	24.4
8	25	0	0.6	No	Yes	50	24.3
9	Sham-group	0	0	No	No	10	14.7
10	Sham-group	0	0	No	No	10	14.7
11	Sham-group	0	0	No	No	10	26.7
	RSPV-LA junction Dose (Gy)	∆Voltage (mV) Endocardial Tag Points RSPV-LA junction	Hyperechoic on TTE/ICE	Macroscopic Lesion	Lesion in Histology	∆Wenckebach end Follow-up (ms)	Follow-up (weeks)
12	40	−1.7*** ††	Yes	Yes	Yes	−5	24.4
13	40	−0.8* ¥¥	Yes	No	Yes	10	24.4
14	30	−0.6* ¶¶	Yes	No	Yes	5	24.6
	LV freewall Dose (Gy)	∆Mean LV-Target Endocardial Voltage (mV)	Abnormal or Double Potentials Endo-/Epicardial (#tags)	Macroscopic Lesion	Lesion in Histology	Hyperechoic on TTE/ICE	Follow-up (weeks)
15	40	−0.5 ‡‡	−/10	Yes	Yes	Yes	12.6
16	40	−0.6 †††	−/−	Yes	Yes	Yes	12.6
17	40	−3.4 *** ¥¥¥	19/-	Yes	Yes	Yes	24.6

Upper row: anmals irradiated at the atrioventricular junction. Middle row: outcome for animals irradiated at the left atrial right superior pulmonary vein junction. Lower row: outcome for animals irradiated at the left ventricular freewall. n.a. 3° AVB = not applicable due to complete atrioventricular block. TTE = transthoracic echocardiography; ICE = intracardiac echocardiography; † = Deceased from device-related infection; ¥ = Complete AV block seventeen weeks after irradiation on surface ECG. ^¶^Lesion misplaced into the posterior left ventricular outflow tract. **∆**Wenckebach describes the difference in the Wenckebach-block cycle length occurrence for the baseline and follow-up study. Two-sided t-test for comparison of mean voltage at the target position at baseline *versus* the mean at follow-up date. *p < 0.05; ***p < 0.0001. The following numbers state n of endocardial tag points included into the statistical test. ^††^n = 518; ^¥¥^ n = 529; ^¶¶^n = 315; ^‡‡^n = 408; ^†††^n = 337; ^¥¥¥^n = 391.
