# Supplementary material for: Feasibility Study on Cardiac Arrhythmia Ablation Using High-Energy Heavy Ion Beams
Source: Sci Rep. 2016 Dec 20;6:38895. doi: 10.1038/srep38895 (PMC5171237; doi:10.1038/srep38895)
Supplement: Supplementary Dataset 1 [file srep38895-s1.doc]

**Supplementary Data:**

**Feasibility Study on Cardiac Arrhythmia Ablation Using**

**High-Energy Heavy Ion Beams**

H. Immo Lehmann^1†^, Christian Graeff^2†^, Palma Simoniello^2^, Anna Constantinescu^2^, Mitsuru Takami^1^, Patrick Lugenbiel^4^, Daniel Richter^2,5^, Anna Eichhorn^2^, Matthias Prall^2^, Robert Kaderka^2^, Fine Fiedler^3^, Stephan Helmbrecht^3^, Claudia Fournier^2^, Nadine Erbeldinger^2^, Ann-Kathrin Rahm^4^, Rasmus Rivinius^4^, Dierk Thomas^4^, Hugo A. Katus^4^, Susan B. Johnson^2^, Kay D. Parker^2^, Jürgen Debus^6^, Samuel J. Asirvatham^1^, Christoph Bert^2,5^, Marco Durante^2,7^,

& Douglas L. Packer^1*^

From the

^1^Mayo Clinic Translational Interventional Electrophysiology Laboratory, Mayo Clinic, Rochester, MN, USA.

^2^Department of Biophysics GSI Helmholtzzentrum für Schwerionenforschung, Darmstadt, Germany.

^3^Helmholtz-Zentrum Dresden-Rossendorf, Institute of Radiation Physics, Dresden, Germany.

^4^Department of Cardiology, University of Heidelberg, Heidelberg, Germany.

^5^Department of Radiation Oncology, Friedrich-Alexander University Erlangen-Nürnberg, Erlangen, Germany.

^6^Heidelberg Ion-Beam Therapy Center (HIT), Heidelberg, Germany.

^7^Trento Institute for Fundamentals Physics Applications (TIFPA-INFN), University of Trento, Trento, Italy.

^†^Contributed equally

**Supplementary Figures**

**Fig. S1: In-Beam PET during Irradiation of the Right Superior Pulmonary Vein-Left Atrium Junction.** a.) Coronal plane of a PET scan from irradiation of the right superior pulmonary vein left atrial junction projected over the coronal plane of the contrast-enhanced treatment planning CT. b.) β^+^-signal decay over a timeframe of 6 minutes.
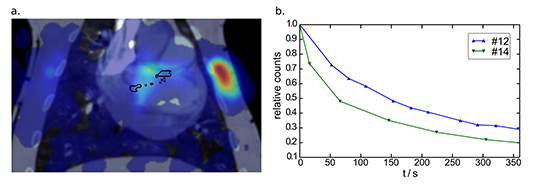


**Fig. S2:** **Animal Position during CT Imaging and Carbon Ion Irradiation.** Animal in the custom fixation device used for imaging and irradiation. A thermoplastic mask was modeled on the chest. The room laser system isocenter (red lines) was marked on the mask by a radio-opaque marker (Beekley Medical; Bristol, CT, USA) and felt pen. The laser lines as well as the borders of the mask were also tattooed on the animal’s skin for later reproduction at the irradiation site.

**
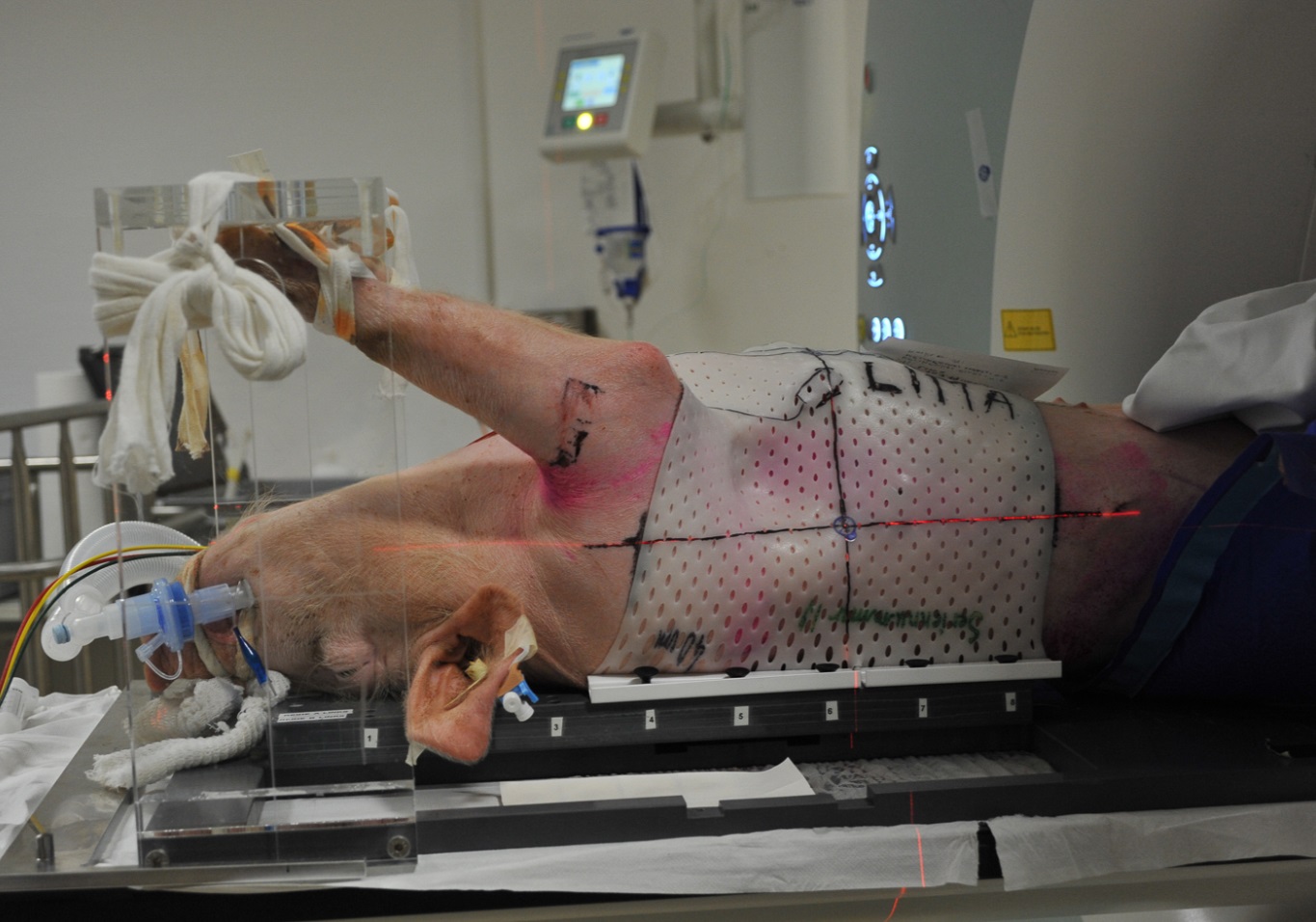
**
